# Comparison of Ophthalmologists versus Dermatologists for the Diagnosis and Management of Periorbital Atypical Pigmented Skin Lesions

**DOI:** 10.3390/jcm13164787

**Published:** 2024-08-14

**Authors:** Giovanni Rubegni, Marco Zeppieri, Linda Tognetti, Elisa Cinotti, Ernesto De Piano, Martina D’Onghia, Matteo Orione, Caterina Gagliano, Tommaso Bacci, Antonio Tarantello, Nicola Lo Russo, Niccolò Castellino, Giusy Miranda, Alessandra Cartocci, Gian Marco Tosi, Teresio Avitabile

**Affiliations:** 1 Ophthalmology Unit, Department of Medicine, Surgery and Neurosciences, University of Siena, 53100 Siena, Italy; giovannirubegni@gmail.com (G.R.);; 2 Department of Ophthalmology, University Hospital of Udine, 33100 Udine, Italy; 3 Department of Medical, Surgical and Neurological Sciences, Dermatology Section, University of Siena, 53100 Siena, Italy; 4 Department of Ophthalmology, University of Catania, 95123 Catania, Italy; 5 Department of Medicine and Surgery, University of Enna “Kore”, 94100 Enna, Italy; 6 Department of Medical Biotechnology, University of Siena, 53100 Siena, Italy

**Keywords:** eyelid skin lesions, dermoscopy, ophthalmoplasty

## Abstract

**Background/Objectives:** Lentigo maligna (LM) and lentigo maligna melanoma (LMM) are significant subtypes of melanoma, with an annual incidence of 1.37 per 100,000 people in the U.S. These skin tumors, often found in photo-exposed areas such as the face, are frequently misdiagnosed, leading to delayed treatment or unnecessary excisions, especially in the elderly. Facial melanocytic skin tumors (lentigo maligna—LM/lentigo maligna melanoma—LMM) and their simulators (solar lentigo, pigmented actinic keratosis, seborrheic keratosis and lichen planus-like keratosis) often affect the periocular region. Thus, their diagnosis and management can involve different medical figures, mainly dermatologists and ophthalmologists. This study aimed to evaluate the ability of ophthalmologists to diagnose and manage pigmented skin lesions of the periorbital area. **Methods**: A multicentric, retrospective, cross-sectional study on a dataset of 79 periorbital pigmented skin lesions with both clinical and dermoscopic images was selected. The images were reviewed by six ophthalmologists and two dermatologists. Descriptive statistics were carried out, and the accuracy, sensitivity, and specificity, with their 95% confidence interval (95% CI), were estimated. **Results:** Ophthalmologists achieved a diagnostic accuracy of 63.50% (95% CI: 58.99–67.85%), while dermatologists achieved 66.50% (95% CI: 58.5–73.8). The sensitivity was lower for ophthalmologists in respect to dermatologists, 33.3% vs. 46.9%, respectively. Concerning the case difficulty rating, ophthalmologists rated as “difficult” 84% of cases, while for dermatologists, it was about 30%. Management was also consistently different, with a “biopsy” decision being suggested in 25.5% of malignant lesions by ophthalmologists compared with 50% of dermatologists. **Conclusions:** Ophthalmologists revealed a good diagnostic potential in the identification of periorbital LMs/LMMs. Given progressive population ageing and the parallel increase in facial/periorbital skin tumors, the opportunity to train new generations of ophthalmologists in the early diagnosis of these neoformations should be considered in the next future, also taking into account the surgical difficulty/complexity of this peculiar facial area.

## 1. Introduction

According to statistics from the Surveillance, Epidemiology, and End Results (SEER) program, lentigo maligna (LM) and lentigo maligna melanoma (LMM), with an annual incidence of 1.37 cases per 100.000 population, is the second most common clinicopath-ologic subtype of melanoma in the United States, preceded by [1]. The importance of the body location—and, consequently, the modality of sun exposure (chronic vs. intermittent)—for the development and prognosis of MM has been pointed out by several studies. Moreover, LM/LMM of the face, scalp, and neck seems to have poorer survival rates compared with MM of other areas of the body. Usually, these skin tumors affect the photo-exposed skin areas, particularly the face, and not infrequently the periocular region [2]. The differential diagnosis of LM/LMM includes several pigmented skin lesions (PSLs) presenting as macules/papules, including solar lentigo (SL), pigmented actinic keratosis (PAK), seborrheic keratosis (SK), and lichen planus-like keratosis (LPLK). LM misdiagnosed as benign pigmented lesions can lead to inappropriate management and delayed melanoma diagnosis. The erroneous diagnosis of an LM-like benign lesion can lead to unnecessary excisions, resulting in surgical morbidity, especially in elderly patients, and avoidable facial cosmetic issues [3,4].

Conditions affecting periorbital regions, including eyelid margin abnormalities, comprise an interdisciplinary problem that is of interest to doctors of various specialties, including ophthalmologists, dermatologists, and plastic surgeons [5,6,7]. For such specialists, the early recognition of malignant tumors is imperative; according to the literature, 5–10% of all skin cancers are located on the periorbital region [8]. Currently, among non-dermatology specialists, the diagnosis of eyelid lesions is based on the patient’s history and clinical features. How successfully ophthalmologists interpret these data and make a clinical diagnosis is, to date, poorly documented [9,10].

Digital photography, total body photography (TBP) (2D or 3D), dermoscopy, reflectance confocal microscopy (RCM), optical coherence tomography (OCT), line-field confocal optical coherence microscopy (LC-OCT) and high-frequency ultrasound (HFUS) are the most commonly used imaging methods for the diagnosis and monitoring of skin cancers. Increasing evidence supporting the efficacy of some of these modern methods in the diagnosis of skin cancer has led to their being incorporated into the recommendations of international guidelines. In particular, dermoscopy is a non-invasive and very low-cost technique in respect to all the abovementioned devices. In a direct naked-eye examination, light is reflected, dispersed, or absorbed by the stratum corneum as a function of its re-fraction index and its optical density, making it impossible to view deeper underlying structures. Dermoscopy, using a hand-held magnification device following the applica-tion of a liquid at the skin–device interface (reducing light reflection), or using cross-polarized instruments, allows the visualization of skin lesions located in the epidermis and upper dermis not seen with the naked eye. Dermoscopy, either analog or digital, represents a cornerstone in dermatological diagnostics. It is frequently used for the diagnosis and monitoring of both pigmented and non-pigmented lesions, MM, and NMSC, helping to achieve an earlier diagnosis. Dermoscopy has been demonstrated to drastically increase the diagnostic accuracy of both benign and malignant skin lesions when compared with naked-eye examination [11,12]. The improvement in diagnostic performance has prompted even non-dermatology specialists to use it successfully [13,14].

There are currently no papers in the literature investigating the diagnostic ability of ophthalmologists, with and without dermoscopy, in the assessment of skin lesions. However, there are several articles on the diagnostic skills of general practitioners [15,16], medical students [17], and other specialists [18,19] in this field.

The aim of this paper was to evaluate the ability of ophthalmologists to diagnose and manage periorbital area lesions, making use of a medical history and clinical and dermoscopic images.

## 2. Materials and Methods

### 2.1. Case Study

**Figure 1:**
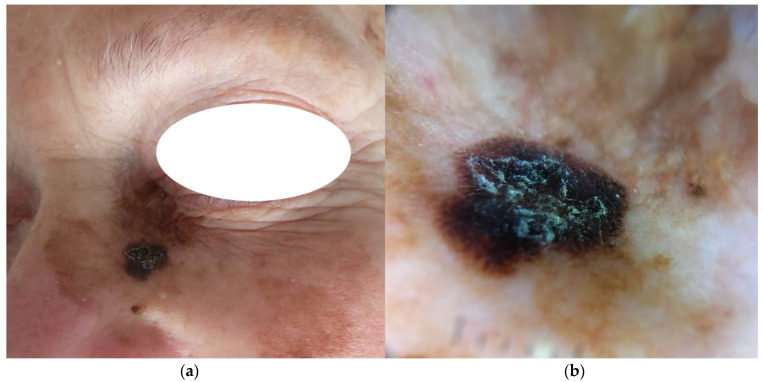
Clinical (**a**) and dermoscopic (**b**) presentation of a lentigo maligna melanoma in the inferior palpebral region. A heavily pigmented and hyperkeratotic area corresponding to the tumoral clone is present in the context of a solar lentigo, over photodamaged skin (**b**), polarized dermoscopy 20×.

This study was carried out in compliance with the Helsinki Declaration; ethical approval was waived because of the retrospective nature of the study, and because all the procedures performed were part of routine care. For this multicentric, retrospective, cross-sectional research, eligible participants were subjects ≥ 18 years old with equivocal periorbital PSLs. A database of 1197 atypical PSLs (the iDScore facial dataset) excised with a clinical suspicion of malignancy was collected [15,16]. Each case was composed of one clinical picture, one standardized dermoscopic image, and four pieces of objective anamnestic data, namely: maximum diameter (mm), patient age (years), patient sex (male/female), and facial site. The face was divided into 6 areas, i.e., orbital area, forehead, nose, cheek, chin and mouth area. This classification was obtained by taking into account anatomical, morphological, and aesthetic features [17]. From the iDScore facial dataset, a subset of periorbital area lesions was derived. This sample was constituted of 79 cases with both clinical and dermoscopic images (Figure 1).

### 2.2. Tele-Diagnostic Test

The images were tested by 6 ophthalmologists (NC, GR, MO, TB, AT, NL); in particular, 2 out 6 had less than 4 years of experience in ophthalmology (GR, MO). Each ophthalmologist evaluated each case, giving an indication of diagnosis (i.e., benign or malignant) and of management (i.e., short follow up, reflectance confocal microscopy, or biopsy/excision). In addition, they assessed the grade of difficulty of the case and the confidence in their diagnosis with the 5-point Likert scale. A total of 474 evaluations (79 cases × 6 ophthalmologists) were obtained. The same test was conducted by an expert dermatologist (LT) and one resident in dermatology (MD), thus obtaining a total of 158 evaluations (79 cases × 2 dermatologists).

### 2.3. Statistical Analysis

Descriptive statistics were carried out: the mean and standard deviation were calculated for quantitative variables, and instead, absolute frequencies and percentages for the qualitative ones. The accuracy, sensitivity, and specificity, with their 95% confidence interval (95% CI), were estimated. A proportion test was used to compare the accuracy, sensitivity, and specificity. All the analyses were carried out with R version 4.1.1.

## 3. Results

The mean age of the 79 patients reviewed was 62.2 ± 12.5 years. Fifty-three (67.1%) patients were female, and 26 (32.9%) patients were male. Thirty-two out 79 lesions (40.5%) were LM/LMM, 8 (10.1%) were PAK, 6 were atypical nevi (7.6%), 26 were solar lentigo (32.9%), 3 were seborrheic keratosis (3.8%), and 4 were seborrheic lichenoid keratosis (5.1%) (see Table 1). In summary, 50.6% of our lesions were malignant, and 49.4% were of a fully benign nature.

Comparing the performance of ophthalmologists with dermatologists, it turned out that they yielded a similar accuracy: *p* = 0.565, 63.50% (95% CI: 58.99–67.85%) vs. 66.50 (95% CI: 58.5–73.8%), respectively. The specificity is comparable between the two specialists (*p* = 0.428), too. Indeed, ophthalmologists obtained a specificity of 84.0% (95% CI: 79.2–88.1%) while dermatologists achieved 79.7% (95% CI: 70.2–87.4%). By contrast, dermatologists obtained a significantly higher sensitivity (p < 0.05), 46.9% (95% CI: 38.3–55.8%) vs. 33.3% (95% CI: 26.7–40.5%). The ophthalmologists assessed the observed cases as difficult/very difficult to classify in approximately 84% of the cases, both for benign and malignant lesions. In contrast, dermatologists classified 30% of benign case evaluations and 35% of malignant case evaluations as difficult/very difficult. The same trend was also observed for confidence in the diagnosis. Ophthalmologists, furthermore, stated themselves to be mildly underconfident/not confident in approximately 80% of evaluations for benign lesions and 85% of malignant lesions. Dermatologists were this in 17% and 25% of cases, respectively (Table 2).

Regarding the lesions’ management, a conservative approach was preferred by dermatologists, with a biopsy in 23.9% of benign cases, versus 14.2% of the ophthalmologists. For the malignant cases, instead, dermatologists suggested excision for about half, versus 25.5% for the ophthalmologists (Table 3).

## 4. Discussion

The primary function of the eyelid is to safeguard the eyeball, despite its relatively small surface area. The eyelid’s position and thin skin often leave it exposed to UV rays and other irritants. This susceptibility, combined with the presence of these agents, increases the risk of eyelid tumor development; it is indeed this area that is one of the most frequent cutaneous tumor locations, accounting for about 5–10% of all skin neoplasms. Given this clinical relevance, the importance of proper framing skills becomes crucial for ophthalmologists. This aspect takes on even greater importance, since interest in the field of ophthalmoplasty has increased considerably in recent years [18,19,20,21,22,23].

Despite the increasing interest of ophthalmologists in periocular skin lesions, data in the literature are scarce to date. Most of the few reports analyze ophthalmologists’ accuracy in cutaneous lesions such as melanocytic nevi, papillomas, epithelial inclusion cists, basal cell carcinoma (BCC), squamous cell carcinoma (SCC), and sebaceous gland carcinomas (SBCs). Abraham et al. reported that, in a group of 116 BCCs diagnosed by the ophthalmology department, only 81 were found to be such upon histological analysis, for a diagnostic accuracy of about 69.8% [24]. Two more recent studies reported an accuracy in identifying malignant lesions of 86% [10] and an accuracy of 98% in diagnosing benign lesions [9]. The better diagnostic accuracy reported by these authors is, in our opinion, related to the easier diagnosis of the examined neoformations [25] In the present study, the performance of ophthalmologists in the diagnosis of difficult atypical PSLs, including LM/LMM of the periorbital region, was evaluated. As a reference, the same lesions were assessed by two dermatologists too. All the lesions were evaluated taking into account a few mandatory morphological parameters (lesion diameter + facial area) and patient anamnestic data (age, sex). It is worth pointing out that, for the first time, a panel of ophthalmologists analyzed dermoscopic images, along with clinical pictures of each lesion.

Another point to underline is that that the case study was composed of atypical PSLs, thus judged “difficult” by a dermatologist expert in the field. Indeed, all of them had been surgically removed/biopsied in order to rule out malignancy. This explains why the examined lesions were “difficult to frame” even by dermatologists, who achieved an accuracy of 66.50% (i.e., a sensibility of 46.9%, specificity of 79.7%). Ophthalmologists revealed a similar accuracy value (63.50%), with a sensibility of 33.3% and a specificity of 84%. Although the diagnostic accuracy was comparable, sensibility values differed significantly (33.3% vs. 46.9%) between the two groups. This indicates that ophthalmologists, compared to dermatologists, are more prone to underdiagnose malignant lesions. Furthermore, it is important to note that, while the results regarding diagnostic ability are similar, the data regarding the rating of diagnostic confidence, lesion difficulty, and, above all, management is consistently different. When evaluating lesions of a benign nature, ophthalmologists considered them to be at least difficult in 82.6% of the cases, and considered themselves very unsafe in 80.8% of the cases. In contrast, dermatologists rated the same lesions as “at least difficult” in 29.6% of the cases, and considered themselves very insecure in 17% of the cases. This important discrepancy is also found in malignant lesions, which ophthalmologists rated as at least difficult in 83.8% and as very unsafe in 86.5% of the cases, whereas dermatologists rated them as at least difficult in 35.8% and as very unsafe in 24.5% of the cases. In our opinion, these results can be explained based on the following items: (i) lesion assessment through the tele-dermoscopic/tele-diagnostic setting may have induced ophthalmologists to underestimate, on average, malignant lesions; (ii) the lack of dermatologic knowledge on skin tumors in ophthalmologists leads them to detect benign features before malignant ones; and (iii) the high number of clinically equivocal cases could have biased the diagnostic confidence and case rating of the ophthalmologists. However, especially for benign lesions, the management decisions of the ophthalmologists did not match with their diagnostic confidence (i.e., they diagnosed more lesions as “benign” than dermatologists, despite the fact that they did not feel confident about the diagnosis). This apparent contradiction in clinical management appears even more paradoxical comparing the management that the two specialists adopted. As concerns the ophthalmologists, biopsy of the lesion was deemed necessary in only 14.2% of benign lesions and 25.5% of malignant ones, compared with 23.9% of benign and 49.1% of malignant in the dermatologists group. The discrepancy here observed could be explained by the fact that the ophthalmologists, compared with dermatologists, generally lack the ability of correctly managing difficult lesions, rather than classifying them as malignant/benign. This “deficit” could be solved with courses aimed at informing ophthalmologists on new dermatological imaging methods, above all dermoscopy, and on the clinical management of lesions. As already reported in the literature for other non-specialists, ophthalmologists could attend a course in dermoscopy, which, as described above, could last between 3 and 6 h [15,16,17,18,19], in which the specialist explains what the main dermoscopic patterns are and how to recognize them. It is well known that each lesion is characterized by certain patterns that are related to the histology of that tissue. Regarding facial skin tumors, 13 main patterns have been identified that characterize pigmented lesions in this area [22]. The last part of the course could then be devoted to discussing clinical cases with the dermatologist where, in addition to the diagnosis of the lesion, its management is assessed, possibly with the help of algorithms that make management more immediate for specialists who face skin lesions less frequently.

Introducing dermoscopy to ophthalmologists could offer several significant benefits, including the following:

- Improved diagnostic accuracy: Ophthalmologists could achieve a higher diagnostic accuracy for various skin conditions, including benign and malignant lesions; this could reduce unnecessary biopsies and referrals, or aid in choosing the appropriate biopsy site;
- Improved early detection of skin cancers, particularly melanoma, by allowing non-specialists to identify suspicious lesions that warrant further investigation or referral to a dermatologist;
- Efficient patient management: Dermoscopy can help non-specialists decide which lesions can be monitored over time, versus those needing immediate action, streamlining patient management and potentially reducing wait times for dermatology appointments;
- Cost-effectiveness: By improving diagnostic accuracy and reducing unnecessary procedures, dermoscopy can be cost-effective for healthcare systems. The early detection of skin cancers can lead to less-invasive treatments and better outcomes, further reducing long-term healthcare costs.

While there are several benefits to introducing dermoscopy to non-specialists, there are also some limitations and challenges to consider:

- Training and proficiency: Dermoscopy requires specialized training to interpret the findings accurately. Non-specialists may not receive sufficient training or may have limited time to develop proficiency, potentially leading to misdiagnosis;
- False sense of security: Inadequate training or experience may lead to overconfidence, where non-specialists might overlook or misinterpret lesions, resulting in missed diagnoses of serious conditions like melanoma;
- Ongoing education: Dermoscopy is a rapidly evolving field. Non-specialists may struggle to keep up with the latest advancements, techniques, and diagnostic criteria without continuous education and training;
- Referral decisions: Determining when to refer a patient to a specialist can be challenging. Non-specialists might either refer too many patients, burdening the specialist services, or too few, potentially missing serious conditions;
- Equipment costs: High-quality dermoscopy equipment can be expensive. Non-specialist practices may find the initial investment prohibitive, especially in resource-limited settings;
- Integration into practice: Successfully integrating dermoscopy into a non-specialist’s practice requires changes to workflows and patient management strategies and possibly additional administrative support, which can be challenging to implement, especially in busy practices with high patient volumes.

As with any newly introduced diagnostic methods, dermoscopy should be used with caution, especially in the early days. However, a dermoscopic photograph of the suspected lesion could be sent to a dermatologist colleague via telemedicine, saving the patient another visit and allowing the less experienced ophthalmologist to learn from their dermatologist colleague’s assessment.

This study has some limitations. A selection bias should be taken int account, as all cases have been retrospectively collected from excised lesions only; data concerning the age of the patient at lesion onset or the timing for lesion evolution/duration were missing, thus not provided to the examinators (either ophthalmologists and dermatologists) for tele-diagnosis, whereas in frontal examination, we take all this information into account; and only one clinical picture and one dermoscopic (polarized light) photograph was available for each case, whereas in clinical practice, we evaluate multiple light exposure/dermoscopic modalities.

## 5. Conclusions

This study shows that ophthalmologists have great diagnostic potential in the identification of malignant lesions of the face. At the same time, it makes clear how a dermoscopic training course on easy lesions, and therefore on difficult lesions, can increase the diagnostic skill of the ophthalmologist, also aimed at identifying the subtype of the benign lesion (SL, SK) or the lesions to be treated because they are considered pre-cancerous (PAK). The opportunity to train new generations of ophthalmologists in this respect should be taken into account in the near future, because of the increase in population ageing and therefore in the number of patients with atypical PFLs. This training implementation would never be an attempt to replace the dermatologist, who has the ability to assess the skin lesion in a broader clinical context, but, rather, an improvement in the capacity to cooperate between the two specialists for the benefit of the patient. In future studies, we hope to investigate the performance of specialists and non-specialists based on their clinical experience and how that performance changes after adequate dermoscopic training, encompassing the characteristics discussed above.

## Figures and Tables

**Table 1:** Sample characteristics.

	Total N = 79
Patient age	62.18 ± 12.49
Patient sex	
Female	53 (67.1%)
Male	26 (32.9%)
Lesion maximum diameter (mm)	10.81 ± 5.75
Histology	
Lentigo maligna/lentigo maligna melanoma	32 (40.5%)
Pigmented actinic keratosis	8 (10.1%)
Atypical nevi	6 (7.6%)
Solar lentigo	26 (32.9%)
Seborrheic keratosis	3 (3.8%)
Seborrheic lichenoid keratosis	4 (5.1%)

**Table 2:** Test results stratified by malignant and benign cases and by ophthalmologists and dermatologists.

	Benign Cases Number of Images = 47		Malignant Cases Number of Images = 32	
	Opht.	Dermat.	Opht.	Dermat.
Number of evaluations	282	94	192	64
Correctly classified cases	237 (84.0%)	75 (79.8%)	64 (33.3%)	30 (46.9%)
Incorrectly classified cases	45 (16.0%)	19 (20.2%)	128 (66.7%)	34 (53.1%)
Grade				
Very easy	0 (0.0%)	9 (9.1%)	0 (0.0%)	4 (5.7%)
Easy	16 (5.7%)	21 (22.7%)	4 (2.1%)	8 (13.2%)
Moderate	33 (11.7%)	36 (38.6%)	27 (14.1%)	29 (45.3%)
Difficult	134 (47.5%)	17 (18.2%)	103 (53.6%)	18 (28.3%)
Very difficult	99 (35.1%)	11 (11.4%)	58 (30.2%)	5 (7.5%)
Confidence				
Very confident	2 (0.7%)	18 (19.3%)	0 (0.0%)	8 (13.2%)
Mildly confident	20 (7.1%)	42 (44.3%)	5 (2.6%)	19 (30.2%)
Uncertain	32 (11.3%)	18 (19.3%)	21 (10.9%)	21 (32.1%)
Mildly underconfident	110 (39.0%)	6 (6.8%)	75 (39.1%)	11 (17%)
Not confident	118 (41.8%)	10 (10.2%)	91 (47.4 %)	5 (7.5%)

**Table 3:** Management of the 79 cases stratified by ophthalmologists and dermatologists.

	Benign Cases Number of Images = 47		Malignant Cases Number of Images = 32	
	Opht.	Dermat.	Opht.	Dermat.
Number of evaluations	282	94	192	64
Management				
Skin biopsy	40 (14.2%)	23 (23.9%)	49 (25.5%)	31 (49.1%)
Reflectance confocal microscopy	97 (34.4%)	19 (20.4%)	72 (37.5%)	12 (18.9%)
Close dermoscopic follow-up	145 (51.4%)	52 (55.7%)	71 (37.0%)	21 (32.0%)

## Data Availability

Data available upon request.
